# The Norwegian national trauma registry: development process and essential data insights

**DOI:** 10.1186/s13049-025-01390-7

**Published:** 2025-05-01

**Authors:** Kjetil Gorseth Ringdal, Kjetil Tengesdal Holm, Olav Røise

**Affiliations:** 1 Norwegian Trauma Registry, Norwegian National Centre on Trauma, Division of Emergencies and Critical Care, Oslo University Hospital, P.O. Box 4950, Nydalen, Oslo, 0424 Norway; 2 Department of Anaesthesiology, Intensive Care and Operating Theatre Services, Division of Emergency and Critical Care Medicine, Vestfold Hospital Trust, P.O. Box 2168, Tønsberg, 3103 Norway; 3 Department of Prehospital Care, Division of Emergency and Critical Care Medicine, Vestfold Hospital Trust, P.O. Box 2168, Tønsberg, 3103 Norway; 4 Research Support Services, Oslo University Hospital, P.O. Box 4950, Nydalen, Oslo, 0424 Norway; 5 Division of Orthopaedics, Oslo University Hospital, P.O. Box 4950, Nydalen, Oslo, 0424 Norway; 6 Institute of Clinical Medicine, Faculty of Medicine, University of Oslo, P.O. Box 1072, Blindern, Oslo, Norway

**Keywords:** Quality improvement, Patient safety, Registries, Surveillance, Trauma

## Abstract

**Background:**

Understanding trauma epidemiology, patient demographics, injury characteristics, and outcomes is essential for optimising trauma systems. The Norwegian Trauma Registry (NTR) monitors and improves the Norwegian Trauma System, setting care standards and overseeing system development. The registry was officially recognised as a national register in 2013. This study outlines the establishment of the population-based national registry and provides an overview of selected data.

**Methods:**

Norway’s trauma system includes trauma centres, acute care hospitals, and prehospital services. The registry collects injury details, clinical outcomes, and patient experiences. Local NTR databases that are linked to a central database are maintained at each hospital, and only certified data registrars can enter and validate data. This enables data linkages across hospitals. The NTR includes potentially severely injured patients but also includes undertriaged patients (defined as severely injured patients who are not met by a trauma team activation upon hospital arrival). Descriptive statistics were used to analyse data from trauma patients registered between 2015 and 2023. Patient-Reported Outcome Measures (PROMs) from 2022 were also assessed.

**Results:**

From 2015 to 2023, 78 275 trauma patients were recorded, with annual patient inclusion rising from 7586 in 2015 to 9759 in 2023. All 38 Norwegian hospitals contributed data in 2023. Median age was 41 years (IQR: 21–62), and 66.5% were men. The highest injury rate was among those aged 15–24 years. Penetrating injuries accounted for 4.6% of cases. Severely injured patients with New Injury Severity Score (NISS) ≥ 16 totalled 16 678 (21.3%), while 10 509 (13.4%) had an Injury Severity Score (ISS) ≥ 16. Polytrauma was identified in 3783 (4.9%) of patients using the Newcastle definition and in 2508 (3.2%) patients using the Berlin definition. In 2023, a trauma team was activated for 8731(89.4%) patients recorded in the registry. PROMs data from 2022 showed that 47.2% (1018/2157) of the patients reported anxiety or depression 12 months post-injury. Among those without physical injuries, 8.0% (11/138) were out of work or education. Of the severely injured patients (NISS ≥ 16) who were employed or in education prior to the injury, 26.4% (83/314) had not returned to work or education after 12 months.

**Conclusions:**

The Norwegian Trauma Registry has been successfully implemented in all trauma hospitals in Norway, enabling comprehensive data collection to support trauma care improvements and research.

**Supplementary Information:**

The online version contains supplementary material available at 10.1186/s13049-025-01390-7.

## Introduction

More than five million people die annually from injuries. Traumatic injuries are the leading cause of death for individuals aged 15–29 years and rank among the top three causes of death and disability for those aged 5–44 years globally [1, 2]. In the WHO European Region, approximately 530 000 people, including nearly 42 000 children and adolescents, died from violence and unintentional injury in 2015 [3]. In Norway, 2045 people died from accidents and suicide in 2022 [4]. Injuries impose significant costs on both individuals and society [1].

While primary prevention is the most cost-effective method for reducing injury-related death and disability, health systems must provide optimal care for injured patients (secondary prevention) [5, 6]. Inclusive trauma systems incorporate high-level trauma centres for the most severely injured and acute care hospitals for less severe cases, involving prehospital services, rehabilitation, community and social care, public health, and commissioners [2]. Historically, regional or state-wide trauma systems centred on major trauma centre have been associated with reduced mortality among severely injured patients [2, 7–13]. However, recent studies suggest that as systems mature, outcome differences between levels of care diminish [7, 14]. Nonetheless, the benefits of higher-level trauma centres may remain more pronounced in cases of severe injury [15, 16], highlighting the importance of continuous evaluation. Improvements in trauma care require detailed knowledge of trauma epidemiology, patient demographics, interventions, clinical outcomes, and the patient journey throughout the treatment chain [17]. Differences in infrastructure, socio-political contexts, geography, healthcare systems, climates, transportation distances, the maturity of pre- and in-hospital trauma systems and the urban-rural mix contribute to variations in trauma systems across countries [18–20]. Given these differences, national and international comparisons and benchmarking of trauma care are crucial for identifying key factors associated with good outcomes [21]. To effectively monitor trauma system quality, populations-based regional and national trauma registries are essential, tracking major trauma care processes and outcomes across the entire trauma system [22]. These registries facilitate hospital and system quality improvement and can be used for benchmarking outcomes through prediction models and assessing process and resource efficiency [23–26]. Additionally, trauma registries support hypothesis generation, study protocol planning, and injury surveillance [27].

Trauma registries should also assess post-hospital treatment phases, including rehabilitation, functional recovery, and return to work or education. However, most registries have traditionally included limited information beyond hospital discharge [5]. Evidence shows that systematically organised trauma systems improve care quality and processes [2, 10, 25, 28].

The Norwegian Trauma Registry (NTR) began full-scale registration in trauma-receiving hospitals in 2015. This paper aims to describe the purpose and establishment of the national, population-based Norwegian Trauma Registry and provides an overview of selected data highlighting key findings.

## Materials and methods

### Study design and case sampling

This paper describes and discusses the establishment of the NTR and utilises anonymous data from the registry to characterise the patient population. Data from patients registered in the NTR between 2015 and 2023 are included. For Patient Reported Outcome Measures (PROMs), only 2022 data are used because PROM registration began in 2021, and 12-month data for incidents in 2023 will not be available until the end of 2024.

Since the NTR had not systematically verified with each hospital whether they conducted systematic searches for undertriaged patients (defined as severely injured patients with ISS ≥ 16 who are not met by a trauma team upon hospital arrival) prior to 2022, undertriage data are presented only for 2023.

### Setting

Norway is one of the least densely populated countries in Europe, covering a total area of 385 000 km^2^ (including Jan Mayen Island and the Svalbard archipelago) with a population of 5.5 million as of 2023 [29, 30]. About 86% of the population resides in more densely populated areas [31], primarily in the south-eastern parts of the country. Norway is a high-income nation with a publicly funded healthcare system.

Norway has implemented a nationwide trauma system comprising four independent health regions (regional health authorities), each with a trauma referral centre. Oslo University Hospital – Ullevål is the only centre that meets the criteria for a Level I trauma centre, as defined by the American College of Surgeons Committee on Trauma [32]. The other trauma centres are comparable to Level II centres. Additionally, there are 34 trauma-receiving acute care hospitals, comparable to Level II or III centres [33, 34].

Advanced prehospital trauma care is provided by 13 anaesthesiologist-staffed ambulance helicopters (HEMS) operating from 12 locations, supported by six anaesthesiologist-staffed Search and Rescue (SAR) helicopters operated by the Royal Norwegian Air Force [35]. Each HEMS and SAR base is equipped with a rapid response car for missions within the base’s vicinity [36]. Additionally, several hospitals in the South-Eastern health region have introduced local anaesthesiologist-staffed rapid response critical care cars, which operate alongside HEMS and SAR.

### Establishment of a national trauma registry in Norway: the process

The establishment of Norway’s national trauma registry was initiated in 2001 by trauma-focused anaesthesiologists and surgeons, supported by the Norwegian Anaesthesiological Society and the Norwegian Surgical Society. This group developed an initial dataset proposal, contributed to the creation of a European trauma core dataset [37], and worked on enhancing the Abbreviated Injury Scale (AIS) [38]. In 2005, the registry was licenced by the Data Inspectorate to collect, store and use person identifiable data without written informed consent, but required patients (or their next of kin) to be informed and given the option to request anonymization. In 2008–2009, Norwegian clinicians contributed to revising the Utstein Template for Documenting and Reporting Data Following Major Trauma [39] and subsequently established a national dataset.

The development of a national web-based medical registration system began in 2006 but was delayed by new regulations and national database processes, finishing in 2014. This system was tailored to manage trauma system complexities, specifically enabling data linkages between hospitals for transferred patients, providing each hospital with access to their data.

**Fig. 1:**
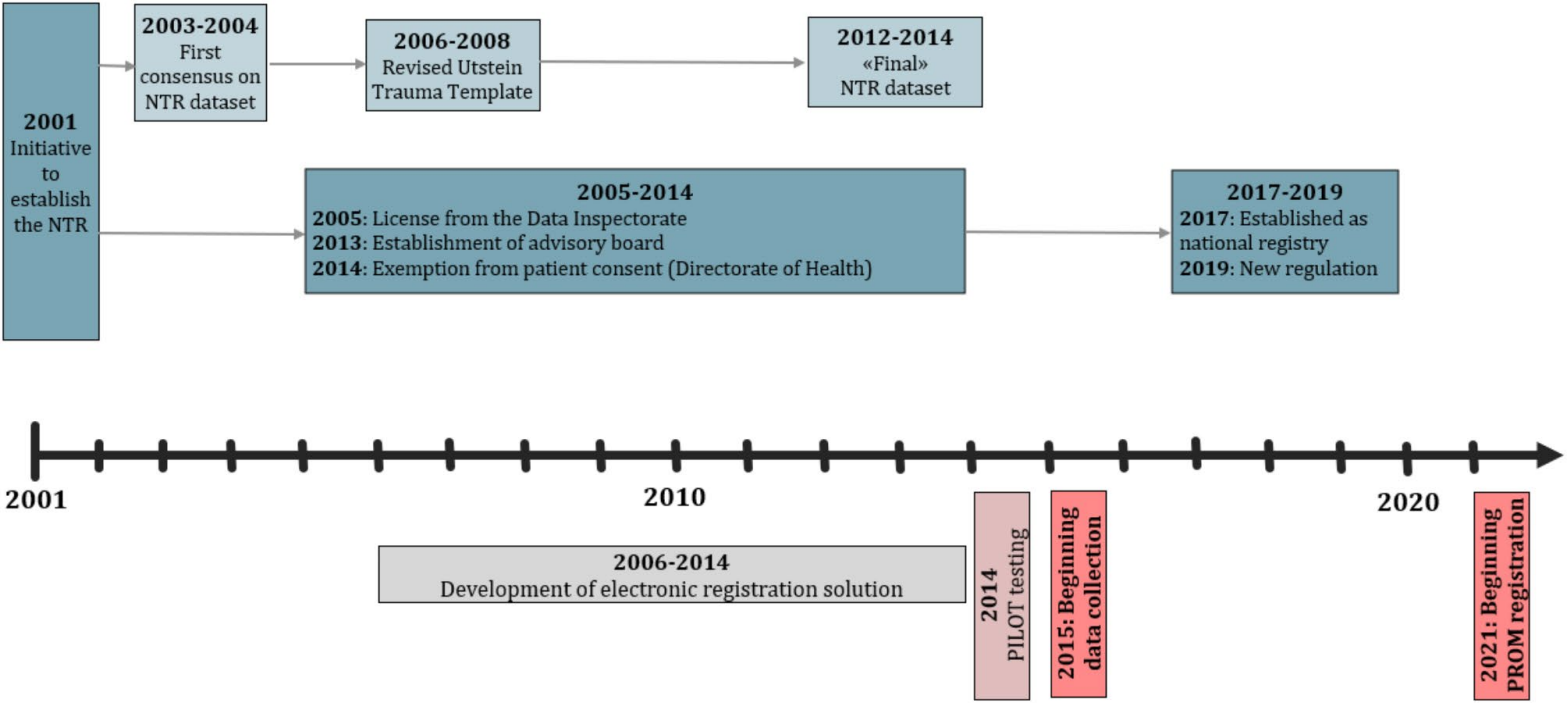
Timeline and development of the Norwegian Trauma Registry (NTR)

In 2013, the Norwegian Directorate of Health resolved to establish a national trauma registry, assigning Oslo University Hospital the responsibility for data management and daily operations. The registry was established as a national population-based medical quality registry. Full-scale registration in trauma-receiving hospitals began in January 2015 (Fig. 1). In 2019, a new regulation from the Norwegian Ministry of Health and Care Services mandated all trauma-receiving hospitals to submit data to the NTR covering the entire major trauma care pathway, including prehospital, hospital care, and rehabilitation [40].

### The database solution

The NTR is a national population-based registry that captures trauma data from the site of injury through the pre- and in-hospital phases to discharge or rehabilitation.

The registration platform provides all trauma-receiving hospitals an online platform for a local trauma registry, legally established as internal quality registries. All local data are instantly stored in a central repository (the national registry) with strict logical and legal separations. Hospitals with self-developed trauma registries can export data if they adhere to the same core set of variables, with uniform definitions, abbreviations and categories for each variable as specified by the NTR (including the revised Utstein Trauma Template data variables). Given that trauma patients may receive treatment in both the prehospital setting and multiple hospital departments before potential transfer to other hospitals, the database is designed to facilitate data entry at any hospital location.

The database allows seamless data entry across prehospital settings and multiple hospital departments, supporting patient transfers between hospitals. Each trauma patient receives a unique NTR ID number that remains consistent across all hospitals where the patient is treated in connection with the specific incident. This ID number enables collection of patient data across multiple hospitals or services.

### Data variables

The database is divided into two sections: trauma data (including accident details, prehospital, emergency department, hospital, and rehabilitation data) and injury scoring data, comprising approximately 170 data variables. The revised Utstein Trauma Template dataset [33, 39] forms the core of the Norwegian data variables [41].

Since 2021, the NTR has also recorded Patient Reported Outcome Measures (PROMs) data. The registry uses the EuroQol 5-Dimension 5-Level (EQ-5D-5 L) [42], along with six additional questions (Additional File 1) [41]. The PROMs forms are sent to all patients over 15 years old who have been involved in an injury/accident. Deceased patients are excluded from receiving the survey. This allows the registry to monitor the patient’s own experience of outcomes after injury. Questionnaires are sent out 6 and 12 months after the accident through www.helsenorge.no. Linkage with the National Population Register, a mandatory national health registry containing information on everyone who resides or has resided in Norway [43], provides 30-day survival data for all patients in the registry.

### Data collection and coding

All trauma-receiving hospitals employ local trauma registry coders (data registrars), usually nurses with trauma experience, who must complete a mandatory NTR coding course and the Abbreviated Injury Scale (AIS) course [44] before being licenced to code. Physicians with trauma care expertise and research backgrounds oversee the local registries. Anatomical injuries are coded according to the AIS© 2005 Update 2008 code set [45]. The Injury Severity Score (ISS) [46] and New Injury Severity Score (NISS) [47] are used as summary measures of overall anatomical injury severity. Severely injured patients are defined as ISS or NISS ≥ 16. Additionally, polytrauma patients are identified using the Newcastle [48–50] and the Berlin definitions of polytrauma [51]. For calculating polytrauma incidence for patients meeting the Berlin definition, we used prehospital GCS values in those cases where patients had been intubated and sedated prior to arrival at the emergency department.

A data definition catalogue, detailing all variables and coding explanations, has been developed [41] and is revised annually to ensure consistency. The NTR Secretariat also provides ongoing support to hospital registrars through guidelines, information letters, and user-support by email and telephone [52, 53].

In 2021/2022, a validation of the coverage rate at the individual patient level, representing 10% of the total population, was conducted at eight of the 38 trauma hospitals. These included a mix of small and large hospitals from across the country. The coverage rate was 100% for patients received with trauma team activation. When undertriaged patients and those with ISS 13 or 14 who were not met by a trauma team (see inclusion criteria, Table 1) were included, the overall coverage rate was 92.2% [54]. In 2022, a data quality study comparing 49 variables registered in the NTR with corresponding electronic patient records in the same 8 hospitals showed excellent data accuracy, though some variables had reduced completeness and require further attention [52]. In 2022/2023, a second validation was conducted at five trauma hospitals, assessing 59 variables, with 48 variables exceeding 90% observed correctness [55].

### Identification and inclusion of trauma patients

The registry’s main purpose is to provide knowledge about the quality and safety of trauma care to ensure that all patients receive equitable and optimal treatment. The NTR includes all patients received by a trauma team upon arrival, regardless of ISS/NISS. It also includes severely injured patients who should have received trauma team activation (TTA) but did not, as well as burn injuries meeting the criteria in Table 1.

**Table 1:** Inclusion and exclusion criteria for the Norwegian trauma registry

Inclusion criteria	Exclusion criteria
1. All patients received by a trauma team upon arrival at the emergency department in trauma centres or trauma-receiving hospitals in Norway, regardless of ISS/NISS	1. Patients with chronic subdural hematoma, without other trauma-related injuries
2. All patients treated at trauma centres and trauma-receiving hospitals in Norway who are not received by a trauma team, but have one or more of the following injuries: • Penetrating injuries to the head, neck, torso, or extremities proximal to the elbow and knee • A single head injury with AIS severity ≥ 3 • NISS > 12	2. Patients involved in drowning accidents, inhalation injuries, and asphyxiation accidents (hanging, strangulation) without other trauma-related injuries¹, as well as hypothermia without other trauma-related injuries
3. All patients who die at the scene of injury or during transport to hospital, who are not delivered to the hospital, but where prehospital management/treatment is initiated	3. Patients who die at the scene of injury without prehospital resources being dispatched

^1^Included if the patient was received by the trauma team upon arrival at a trauma centre or trauma-receiving hospital

AIS: Abbreviated Injury Scale

ISS: Injury Severity Score

NISS: New Injury Severity Score

**Fig. 2:**
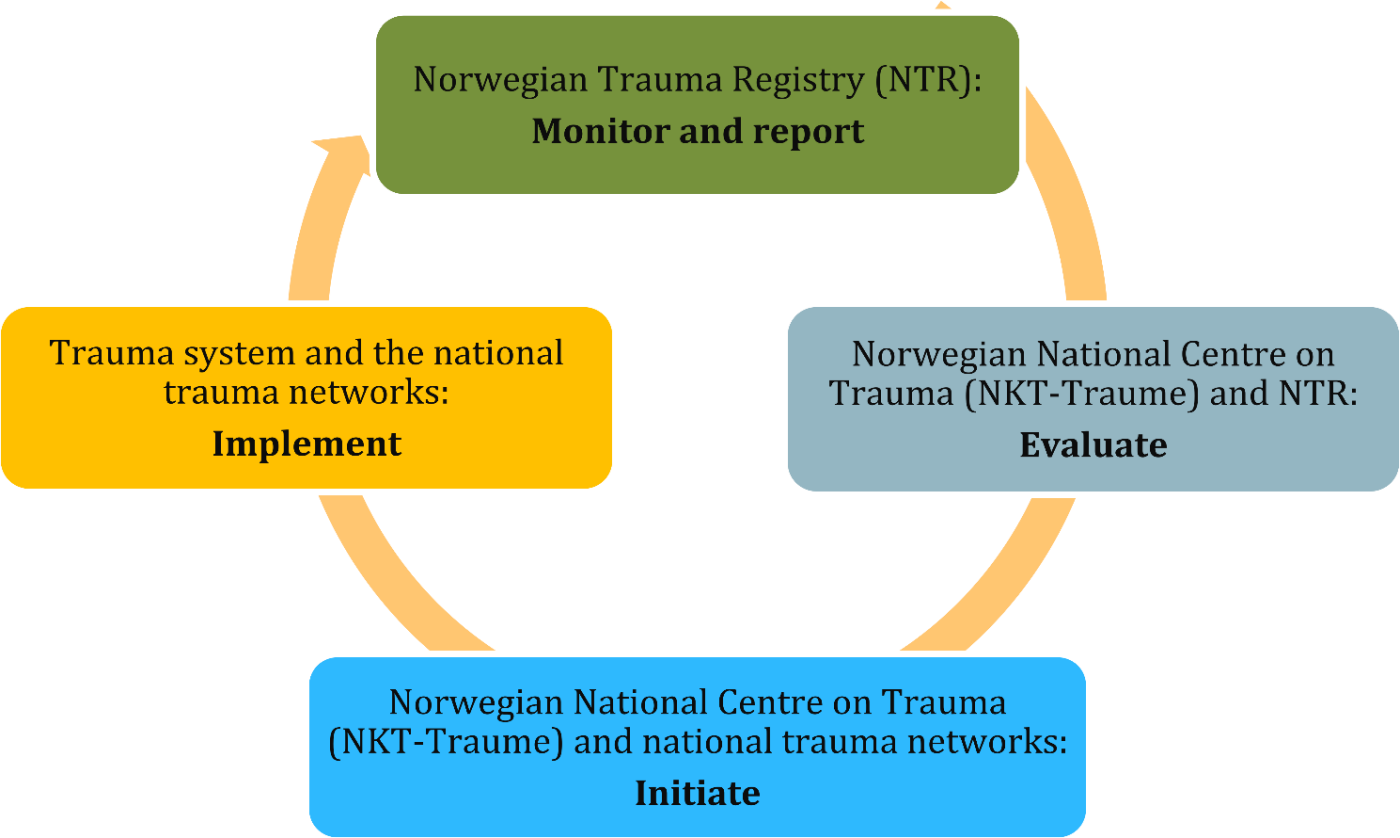
The components of the Norwegian Trauma System

To reduce variation in TTA criteria across Norwegian hospitals [56], the Norwegian Trauma System (Fig. 2) recommended a standardised set of TTA criteria in 2017 [57], revised in 2023. These criteria were based on guidelines from the American College of Surgeons Committee on Trauma [32] and the Field Triage Guideline Recommendations of the U.S. Centers of Disease Control and Prevention [58].

Identifying severely injured patients eligible for inclusion in the NTR, who are not met by a dedicated trauma team can be challenging. Thus, the registry developed search and identification criteria using emergency dispatch centre logs, emergency department records, and diagnosis codes from the hospital’s patient administration system.

### Indicators of the quality and safety of trauma care

Trauma mortality and preventable death rates are currently low in many high-income countries [59], reducing their effectiveness as major performance indicators [12]. Gruen et al. suggest that further improvements in trauma care are unlikely to significantly reduce mortality [12]. Instead, focusing on care quality and patient safety indicators can help identify potential areas for improvement in pre- and in-hospital care, functional outcomes, survivors’ quality of life, and mortality [60, 61]. There are no evidence-based national or international quality indicators within the field of traumatology, but several international trauma systems have defined their own quality indicators, primarily process indicators (procedures and timelines). The NTR has developed a set of indicators based on the National Trauma Plan [62] and Advanced Trauma Life Support principles [63], which are considered significant for ensuring both the quality and safety of trauma care and outcomes (Table 2). The steering committee has defined criteria for acceptable, moderately acceptable, and unacceptable quality.

**Table 2:** Indicators of the quality and safety of trauma care

• Proportion of complete trauma registrations completed within 3 months • Proportion of undertriage (recommended < 5%) • Time from EMCC-call until ambulance personnel arrive at the scene of the incident* • Proportion of patients with GCS < 9 and prehospital airway management • Proportion of patients with GCS < 9 received by the trauma team who are intubated in the emergency room • Proportion of patients with GCS < 9 and ISS ≥ 16 received by the trauma team who are intubated in the emergency room • Proportion of trauma patients received by the trauma team who undergo a chest X-ray during trauma admission • Proportion of trauma patients with severe injury (ISS ≥ 16) received by the trauma team who undergo a chest X-ray during trauma admission • Proportion of trauma patients received by the trauma team who undergo a pelvic X-ray during trauma admission • Proportion of trauma patients with severe injury (ISS ≥ 16) received by the trauma team who undergo a pelvic X-ray in during trauma admission • Proportion of trauma patients received by the trauma team who undergo a CT scan during trauma admission • Proportion of trauma patients with severe injury (ISS ≥ 16) received by the trauma team who undergo a CT scan during trauma admission • Proportion of trauma patients with ISS < 4 received by the trauma team and undergo a CT scan in connection with trauma admission • *Survival 30 days after injury • Survival 30 days after injury for patients with ISS ≥ 16 and ISS < 16	

EMCC: Emergency Medical Communication Centre

GCS: Glasgow Coma Scale

ISS: Injury Severity Score

Undertriage = severely injured patients (ISS ≥ 16) received without trauma team activation / all severely injured patients (ISS ≥ 16) regardless of whether they were received by the trauma team or not

ISS is calculated by summing the squares of the highest Abbreviated Injury Scale (AIS) scores from the three most severely injured AIS body regions

*National quality indicator

The NTR is one of few, if not the only, national trauma registries that collects data on undertriaged patients (i.e., severely injured patients (ISS ≥ 16) who are not met by a trauma team upon hospital arrival).

The results, presented annually to the health regions, show the distribution of hospital performance and act as an incentive for improving quality and safety.

### Organisation and governance structure

The Chief Executive Officer of Oslo University Hospital serves as the data controller (in accordance with the EU General Data Protection Regulation), with the registry acting as the data processor. The formal responsibility, including data processing, lies with the data controller, but a data protection officer ensures that the data processor fulfils its formal responsibilities.

The registry has an academic council (steering committee) representing the regional health authorities, trauma-treating medical specialties, patient organisations, and data registrars. The council guides registry strategies and policies, monitors data collection and quality; and reviews data requests, reports, and publications, ensuring that timelines are met, objectives are clear, and that the interests of health services and regions are addressed.

Furthermore, the registry has established a reference group (advisory body) that offers strategic advice, including socio-economic perspectives to the managing office and academic council, though its advice is not binding.

The registry has regulations for researchers seeking secure access to patient data, ensuring compliance with privacy perspectives, applicable laws and regulations. All medical and health research projects using patient identifiers require approval from a Regional Committee on Medical and Health Research Ethics.

The NTR is supported by the Norwegian Advisory Unit for Medical Quality Registries [64], which assists with design, development, legal issues, data analysis, presentation of results, and use of the registry for clinical improvement. It also assesses and ensures that national registries meet quality standards.

### Statistical analysis

The dataset was extracted from the database on 07.07.2024. The Clopper-Pearson method was used to calculate the confidence interval for a fraction. Unlike some other methods, this approach provides a more conservative estimate, which results in a confidence level exceeding 95%. Continuous data were presented as medians and interquartile ranges (IQRs) due to the non-normal distribution. Normality was assessed for gender, ISS, and NISS using histograms, Q-Q plots, and the Anderson-Darling test, confirming that normality was not met, justifying non-parametric methods.

Statistical analysis was performed using R, version 4.4.0 (The R Foundation for Statistical Computing, Vienna, Austria).

### Ethics declarations

The data used in this project are anonymous and therefore fall outside the mandate of the Regional Ethics Committee. While the data have been recorded and stored without written consent, patients (or their next of kin) are informed of their right to request anonymisation. This information is provided by the treating hospital [65]. The Norwegian Trauma Registry has permission from the Norwegian Data Inspectorate to collect, store, and analyse data confidentially without patient consent. This is in accordance with Norwegian Data Protection regulations (reference number: 03/00058 − 20/CGN) and EU data protection rules. Registry data can be used for quality assessment and research to improve the overall treatment of seriously injured patients.

## Results

### Patient and injury characteristics

**Fig. 3:**
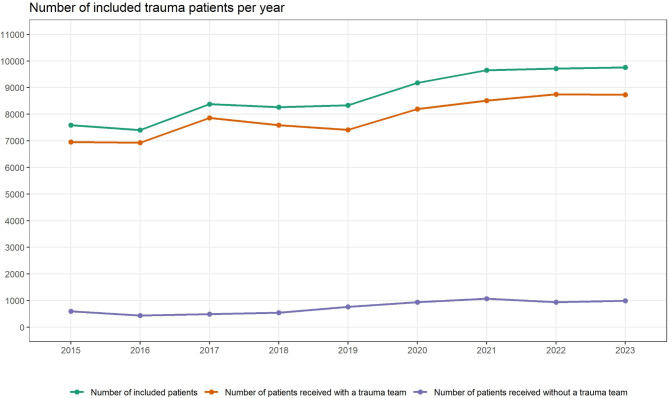
Number of patients per year, including those received with and without a trauma team, since the initiation of data registration in the Norwegian Trauma Registry in 2015

As of December 31, 2023, a total of 78 275 trauma patients have been included in the registry since data collection began in 2015 (Fig. 3), of whom 52 084 (66.5%) were men. The number of patients included has increased annually since the first registrations in 2015, from 7586 patients in 2015 to 9759 patients in 2023.

**Fig. 4:**
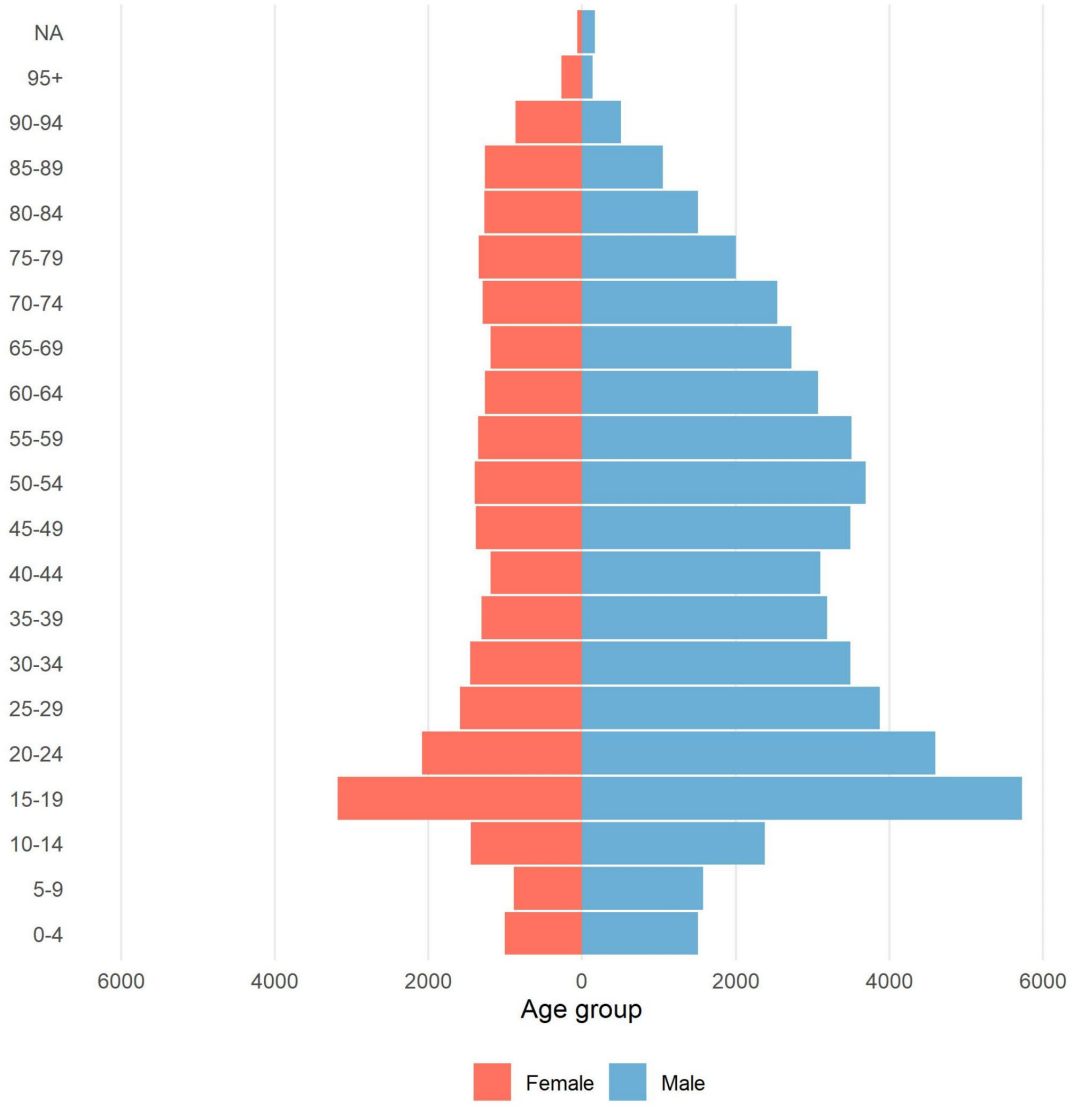
Number of injuries distributed among patients in 5-year age intervals, categorised by gender, as recorded in the Norwegian Trauma Registry from 2015 to 2023

Median age of recorded patients at admission was 41 years (IQR: 21–62). The majority of injured patients from 2015 to 2023 were in the age group 15–24 years (Fig. 4).

Penetrating injuries accounted for 3614 (4.6%) of all 78 275 injuries during this period, irrespective of whether a trauma team was activated at the emergency department during the patient’s admission. This proportion has remained consistent over the years.

The median NISS for the entire population was 5 (IQR: 1–13), whereas the median ISS was 5 (IQR: 1–10). The number (%) of severely injured patients, defined by a NISS ≥ 16, was 16 678 (21.3%), while the number (%) of severely injured patients defined by an ISS ≥ 16, was 10 509 (13.4%). For patients with a NISS ≥ 16, the median NISS was 27 (IQR: 25–34), and for those with ISS ≥ 16, the median ISS was 21 (IQR: 17–26).

The number (%) of polytrauma patients meeting the Newcastle definition was 3783 (4.9%), while 2508 (3.2%) patients met the Berlin definition.

The mortality rate was 4.4% in 2022 and 4.0% in 2023.

**Fig. 5:**
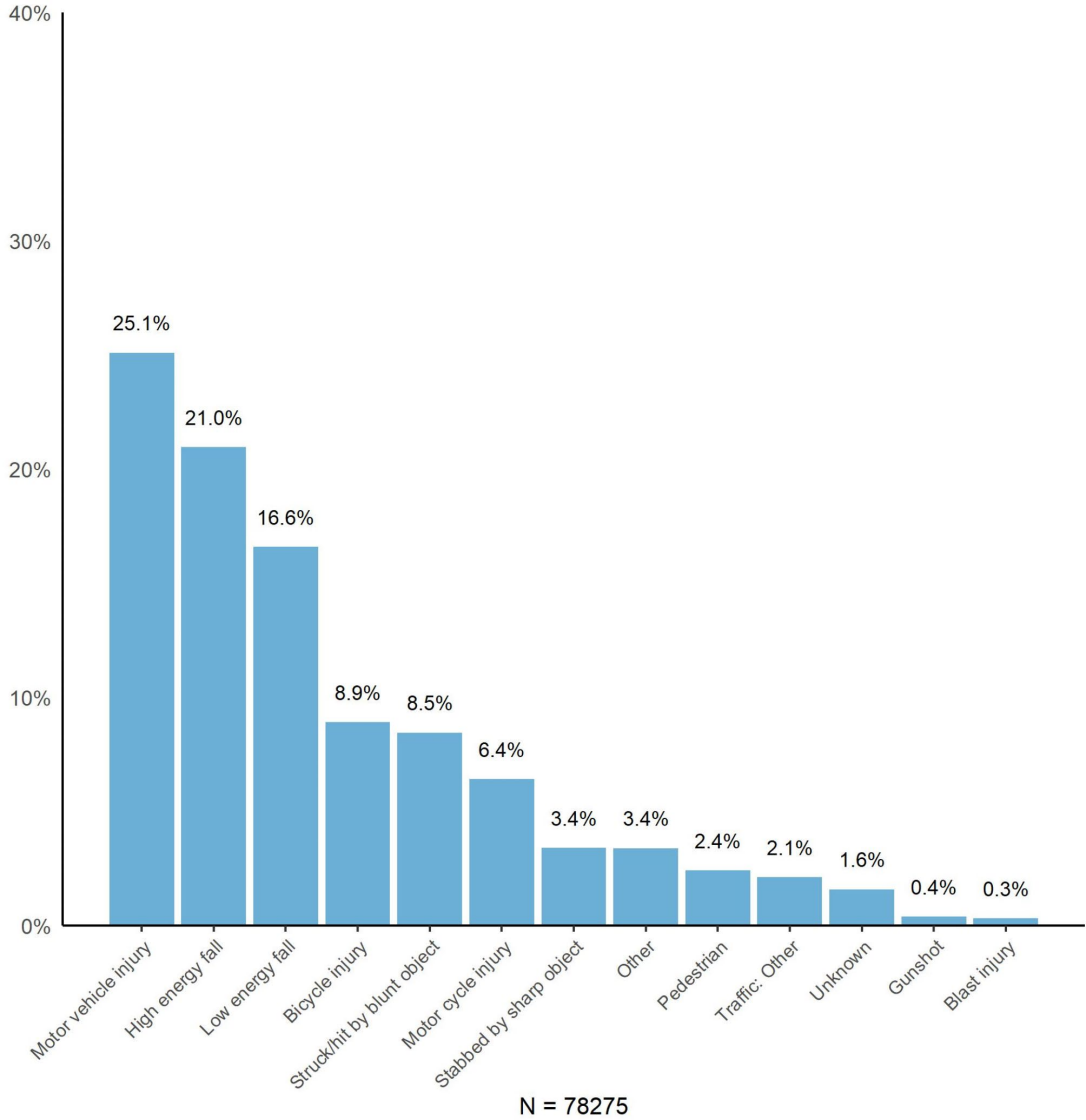
Distribution of primary injury mechanisms for trauma patients recorded in the Norwegian Trauma Registry from 2015–2023. The categories are equal to those in the Utstein Trauma Template dataset

Accidents involving motor vehicles (*n* = 19 643, 25.1%), high-energy falls (*n* = 16 407, 21.0%), and low-energy falls (*n* = 12 984, 16.6%) accounted for the majority (62.6%) of trauma incidents between 2015 and 2023 (Fig. 5).

**Fig. 6:**
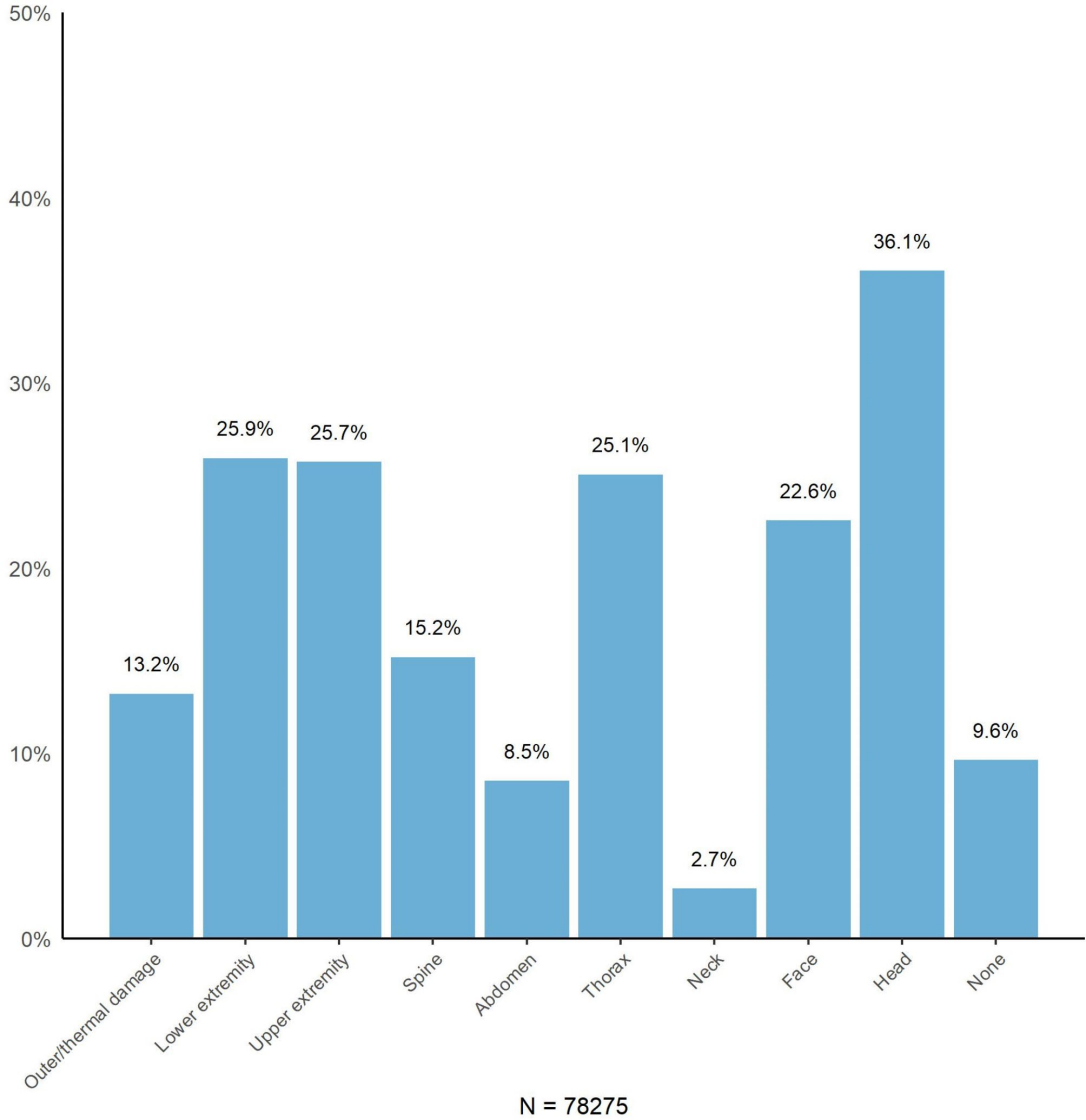
Proportion of patients recorded in the Norwegian Trauma Registry from 2015–2023 with injuries in the different body regions. As patients may have injuries in multiple body regions, the percentages will not sum to 100%. The categories correspond to the body regions of the Abbreviated Injury Scale and are independent of the severity of injury

Head injuries are the most common, affecting 36.1% of patients, followed by injuries to the lower extremities (25.9%), upper extremities (25.7%), and thorax (25.1%) (Fig. 6). However, when extremity injuries are considered as a single category, they are the most frequent overall.

### Undertriage

A total of 8731 (89.5%) TTA were recorded in 2023.

**Fig. 7:**
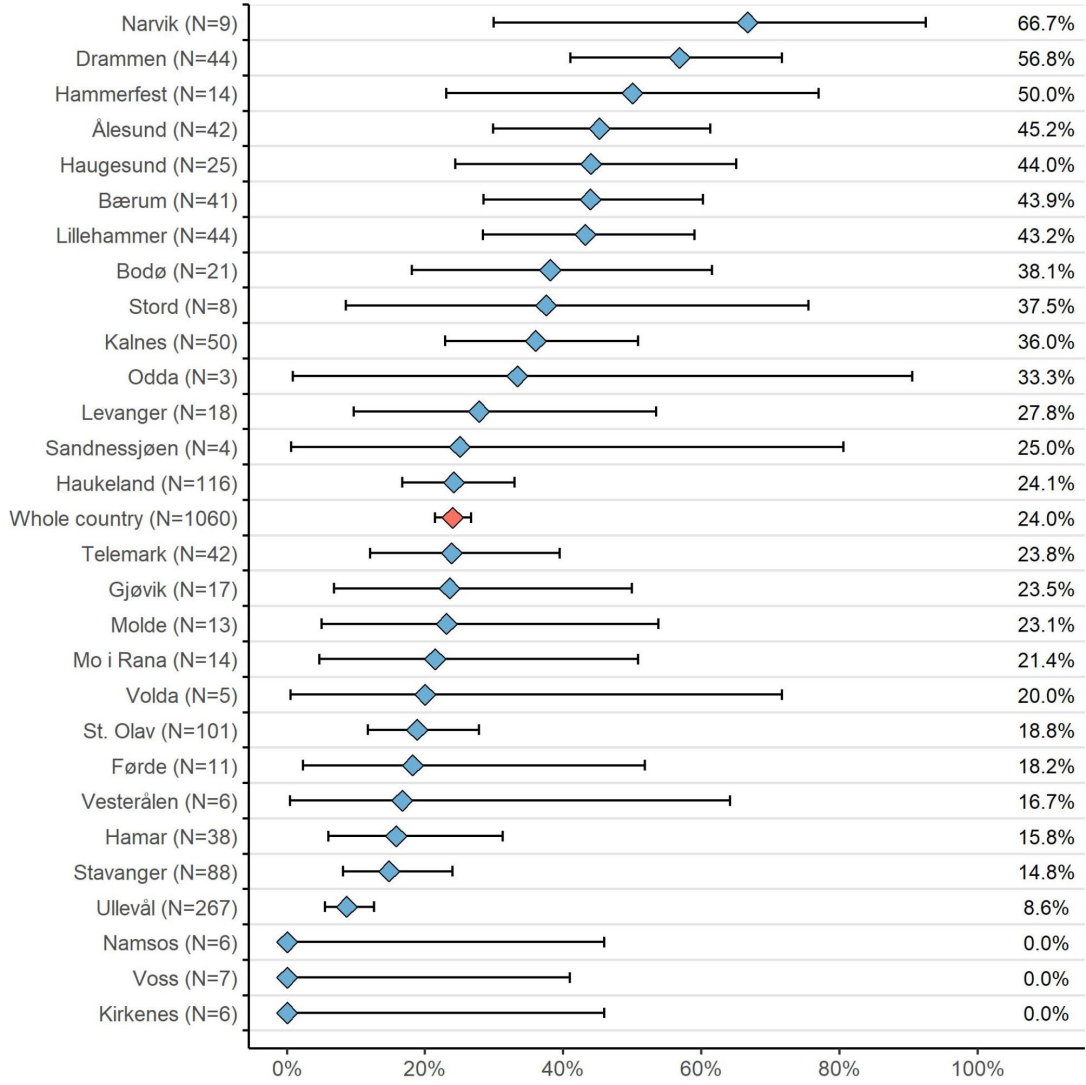
Proportion of undertriaged patients, defined as those with very severe injuries (ISS ≥ 16) who were not received by a trauma team upon arrival at the first hospital, as recorded in the Norwegian Trauma Registry in 2023. Patients who arrived at the hospital more than 48 h after the injury, such as those coming from hospital stays abroad or those seeking medical attention more than 48 h after the accident, were excluded. The figure shows the results only for hospitals that systematically searched for undertriaged patients. The dots in the figure represent the hospital results for 2023, and the outer limits represent a 95% confidence interval

In 2023, 254 of 1060 (24.0%) severely injured patients were undertriaged (Fig. 7). The undertriage rate was higher among women than men, at 29.7% versus 21.7%, respectively. Among undertriaged patients, the mortality rate was 15%, with 38 of the 254 patients dying in 2023. Of these, 95% (36 out of 38) were older than 64 years. Additionally, 37 of the 38 (97%) deceased and undertriaged patients in the registry had sustained injuries from low-energy falls. Among the 230 deceased patients in the registry, 71.2% were over 65 years old and had low energy falls as the mechanism of injury.

### PROM findings

During 2022, PROMs questionnaires were sent to 4916 patients, resulting in 2614 responses (53.2%) received six months after the injury. A further 5822 questionnaires were distributed 12 months later, yielding 2297 responses (39.5%).

**Fig. 8:**
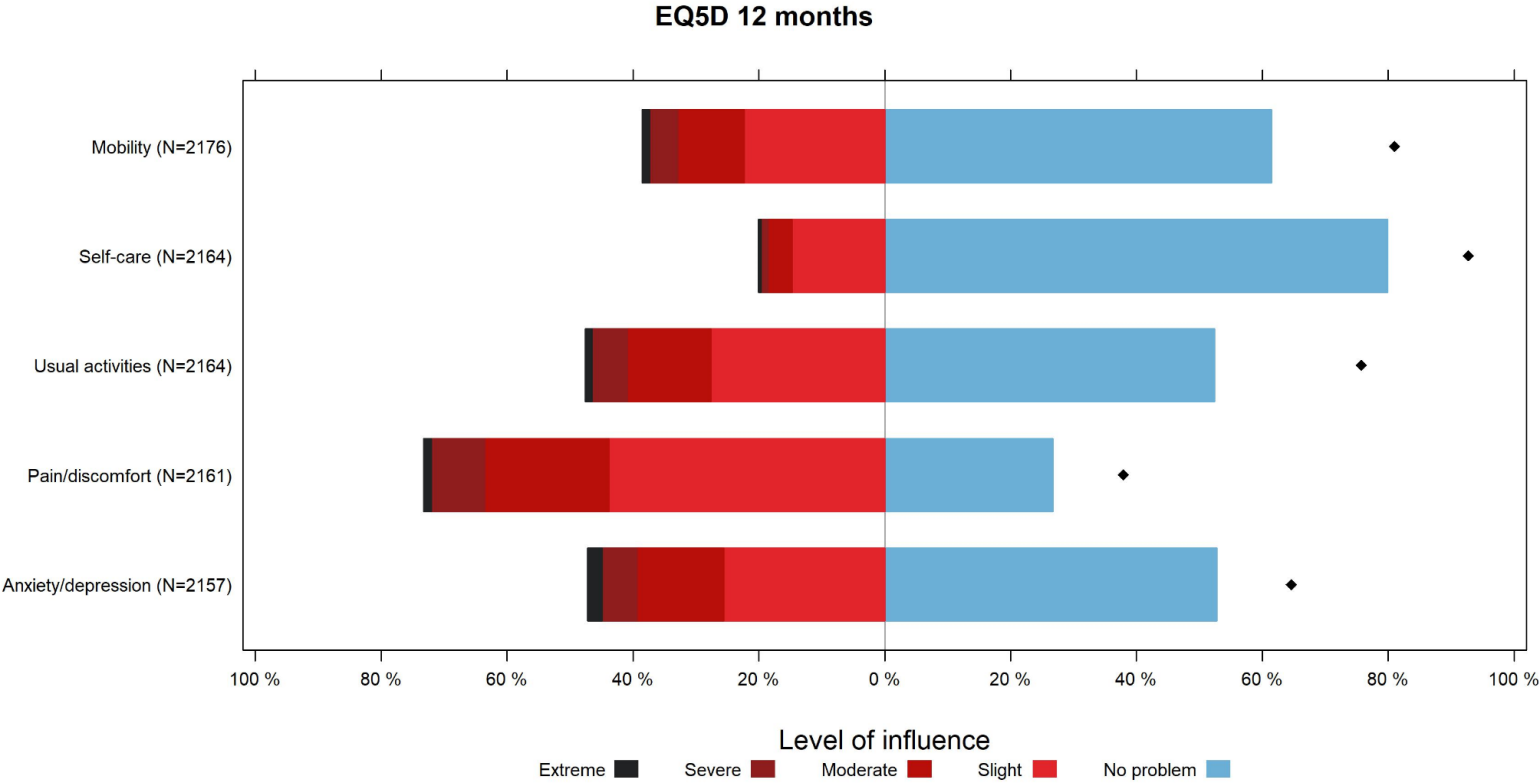
Self-reported health status of trauma patients across the five EQ-5D-5 L dimensions 12 months after the trauma incident, as recorded in the Norwegian Trauma Registry in 2022. The black dots on the right side of the figure represents the proportion of the national norm population who reported that they had no problems (‘No problem’) [66]

Twelve months after the incident, 38.6% (839/2176) of respondents reported mobility issues (Fig. 8). Additionally, 47.2% (1018/2157) of patients experienced some degree of anxiety or depression at 12 months (Fig. 8).

**Fig. 9:**
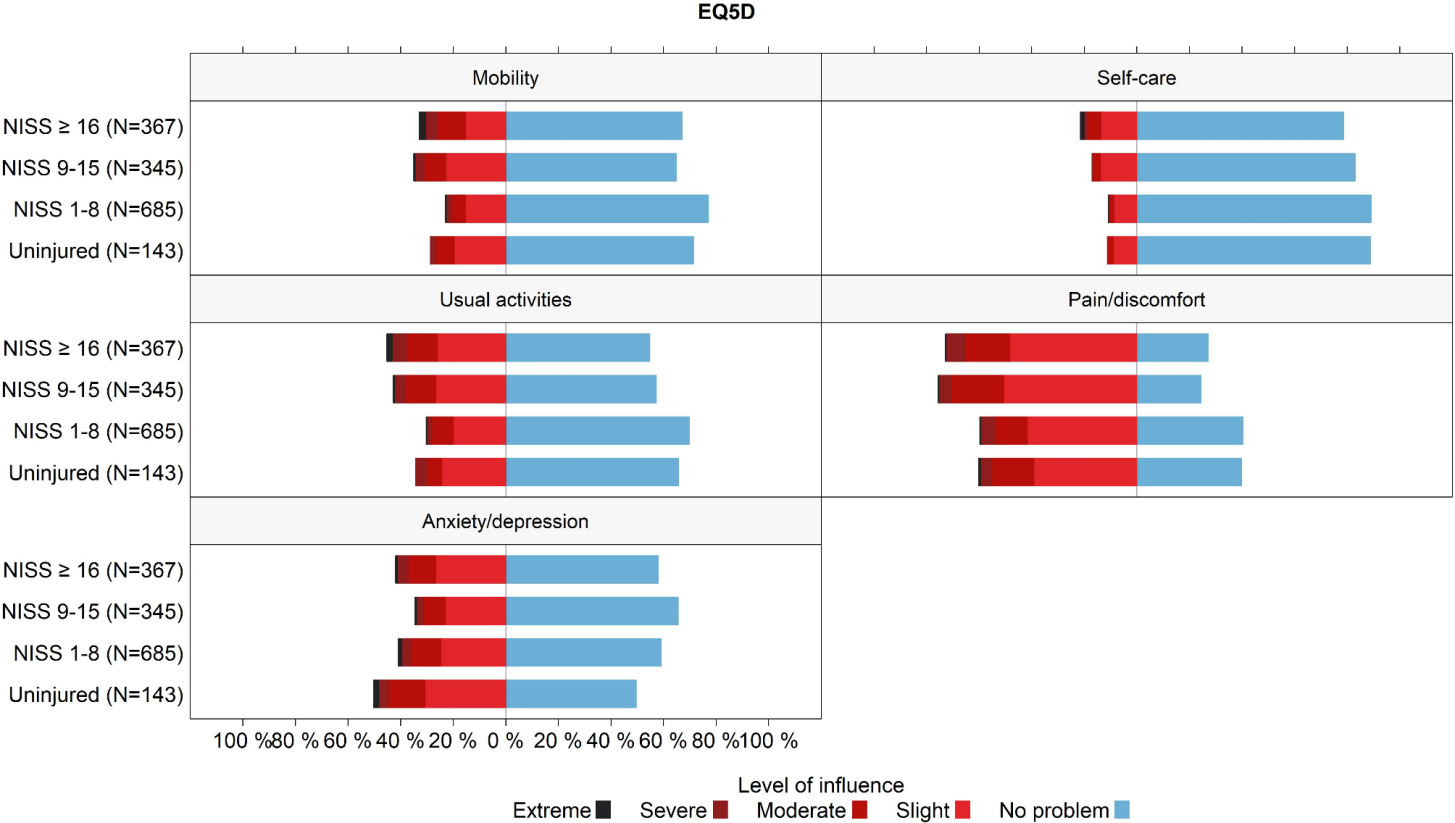
Self-reported health status of trauma patients across the five EQ-5D-5 L dimensions, 12 months after trauma, categorised by NISS groups, as recorded in the Norwegian Trauma Registry in 2022. Patients with long-term (at least one year) illnesses, injuries, or disorders prior to the incident were excluded from this particular analysis

Interestingly, even uninjured individuals reported anxiety and depression (Fig. 9), though further investigation is needed to determine the nature and significance of this finding.

**Fig. 10:**
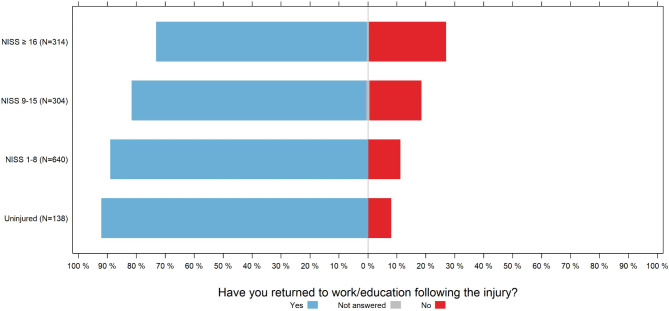
Proportion of patients back in work or education 12 months after the trauma among those who reported being employed or in education before the accident, categorised by NISS category, as recorded in the Norwegian Trauma Registry in 2022

Among those who had been employed or in education prior to the injury, 26.4% (83/314) of the severely injured individuals (NISS ≥ 16) had not returned 12 months later (Fig. 10). Among those who did not sustain physical injuries, 8.0% (11/138) reported that they were not in work or education 12 months after the accident (Fig. 10).

## Discussion

After an extensive start-up phase, the Norwegian Trauma Registry has successfully implemented electronic data collection across all trauma-receiving hospitals in Norway, using a national dataset with a standardised data definition catalogue. The registry has established inclusion criteria, training and support system for data registrars, and clinical indicators to evaluate the performance and safety of trauma care and related outcomes. Additionally, the collection of PROMs data has been integrated into the registry.

The Norwegian Trauma Registry plays a central role in the national trauma system by providing essential data for evaluating and improving the quality and safety of trauma care. While a trauma system can exist without a registry, the registry ensures systematic data collection, which is crucial for the ongoing assessment and enhancement of national trauma care.

Norway is one of the few countries that mandate, by regulation, the inclusion of all trauma patients in a national medical quality registry. Similarly, in the Netherlands, the Dutch Nationwide Trauma Registry is required by government order to capture all acute trauma admissions [67, 68]. These regulatory frameworks facilitate comprehensive data collection, including cases of undertriage. Additionally, the integration of data across the entire trauma care chain using a unique trauma ID number appears to be a distinctive feature of the NTR.

During the initial years, not all hospitals identified or reported all patients eligible for inclusion according to the specified inclusion criteria. Over the years from 2015, the registry has evolved, with significant improvements leading to increased hospital and patient coverage. Compared to the initial years, it may seem as if a higher proportion of severely injured patients received with a TTA died in 2023. Several factors contribute to this trend. One reason is that the registry began to systematise the work of identifying undertriaged patients in 2018. Prior to this, several severely injured patients were not admitted with a TTA, which may have led to their underrepresentation in the registry. As triage practices have improved, a greater number of patients with low energy falls but poor prognoses are also admitted with a trauma team. This shift has resulted in an increase in the number of severely injured patients recorded in the registry, which may help explain the higher observed mortality among those received with a TTA. Therefore, the rise in mortality does not necessarily indicate a decline in care quality but rather reflects more accurate identification and inclusion of severely injured patients. Another possible factor is that more severely injured elderly with low-energy falls may have been admitted by a trauma team; however, this is not confirmed by the data used in this manuscript. Additionally, an improved identification of undertriaged patients from the patient administrative system may also account for this increase.

The relatively low median values for ISS and NISS in this dataset reflect the broad inclusion criteria of the Norwegian Trauma Registry (NTR). These criteria provide a comprehensive overview of all patients admitted with TTA, regardless of injury severity. Including patients who are not classified as severely injured enables the registry to monitor resource utilisation and evaluate the care provided to the entire trauma team population, rather than focusing solely on the most critically injured. Additionally, the NTR includes undertriaged patients (severely injured patients and patients with NISS 13 and 14 not met by a TTA), achieving an inclusion rate of 92.2%. While this is not a perfect capture rate, it ensures that the majority of severely injured patients are represented in the dataset. The remaining 7.8% patients are unlikely to significantly alter the overall distribution of injury severity, as the dataset would still predominantly consist of patients with minor to moderate injuries. This slight omission may marginally affect the median ISS and NISS values, but it does not substantially alter the overall picture. The dataset is therefore characterised by a predominance of patients with minor to moderate injuries, which contributes to the lower median scores. This distribution reflects the comprehensive nature of the registry and its commitment to capturing the full spectrum of trauma patients, ensuring both patient safety, resource monitoring and quality improvement across all levels of injury severity.

The low proportion of patients meeting the Newcastle or Berlin criteria for polytrauma contrasts with findings from other registries, such as the New South Wales Trauma Registry, where 29% of patients meet the Newcastle criteria [50]. Several factors may explain this difference, particularly variations in how trauma patients are identified for inclusion and how TTA criteria are applied across hospitals. While almost all Norwegian hospitals adhere to the national TTA criteria [69] and include all patients meeting the inclusion criteria, other national trauma registries, such as the German Trauma Registry (TR-DGU), the National Major Trauma Registry (NMTR), which collects data for England, Wales, Northern Ireland, and Ireland, and the Australian New Zealand Trauma Registry (ANZTR), use different inclusion thresholds. Consequently, substantial differences exist in which patients are captured by different registries. As high-energy mechanisms trigger TTA, approximately 10% of patients in our registry did not sustain physical injuries, which contributes to the low proportion of polytrauma cases.

A risk of using unvalidated trauma registry data is that incorrect conclusions may be drawn, potentially leading to health care services being governed by data of insufficient quality. To address this, the NTR conducted an evaluation of patient coverage, data completeness, and data accuracy [52]. This evaluation showed that, with trained certified data registrars and robust support tools providing clear descriptions of data variables, the NTR maintains very high data quality on key data variables.

Brohi described trauma registries as a continuous prospective cohort of the trauma population that captures demographics, injury details, process measures, and outcome data [70]. We concur with the author that not all injured patients can be included in a trauma registry, and that trade-offs must be made between inclusion criteria, the number of data points captured per patient, and the intended use of the data. While we acknowledge these limitations, linking data from multiple Norwegian health registries provide an opportunity to compensate for some of these trade-offs by offering a broader perspective on injury patterns and outcomes. By leveraging the national identification number for Norwegian citizens as a key, we can access a wealth of unique and valuable data that can be used for injury surveillance, injury prevention, and trauma system improvements in Norway. For instance, a US study used trauma registry data to identify that a significant percentage of injured children and adolescents were not using proven effective injury prevention devices at the time of injury. These findings highlighted areas for further research and informed local community injury prevention initiatives [71]. Similarly, registry data have been instrumental in optimising pre-hospital triage criteria, contributing to improved trauma system efficiency [72].

Recent findings by Meakes and coworkers from the Sydney area found similar long-term impairments in physical and mental health following polytrauma [73]. Their study highlighted significant decreases in physical health scores at six- and 12-months post-injury, with factors such as prolonged hospital stays, and low initial GCS linked to worse outcomes. These findings align with our own observations, emphasizing the need for targeted follow-up care and further research to identify modifiable predictors of recovery. Such research can provide insights that help drive international collaboration and benchmarking, leading to the improvement of trauma care systems worldwide.

The registry does have some limitations. First, as with all national medical quality registries, there is a risk that data may not be easily accessible for quality assessment at local trauma-receiving hospitals. Although the entered data are immediately accessible upon entry without restrictions, some hospitals encounter difficulties in extracting their own data. However, they can obtain guidance from the NTR secretariate to resolve these issues. This represents a barrier to efficient and timely quality assessment. Hospitals can request data from the NTR, but resource constraints within the NTR management limit this support. Additionally, hospitals involved in the care of a specific trauma patient have, until recently, only been allowed to access their own local data, not to data from other hospitals, even if they were part of the same treatment chain. This limitation may hinder a comprehensive understanding of trauma care issues and impedes quality improvement efforts. However, newly introduced in-hospital electronic patient records now enable involved personnel to gain a read-only access to patient records at the receiving hospital. Second, identifying severely injured patients eligible for inclusion in the NTR, who are not initially met and managed by a dedicated trauma team, can be challenging. The registry has developed a set of search and identification criteria based on logs from the Emergency Medical Communication Centre (EMCC), the emergency department, and diagnosis codes in the hospital’s patient administration system. Despite that, many trauma-receiving hospitals have found these search criteria difficult to implement. Small hospitals with few patients perform weekly reviews of all admissions to ensure that undertriaged patients are identified. This process may be more challenging to implement in larger hospitals. Currently, 28 hospitals systematically search for undertriaged patients; seven conduct searches, but not in a fully systematic manner, while three do not search for these patients at all [55]. Third, one of the inclusion criteria of the registry is prehospital trauma deaths, but we do not have complete data, partly because we do not have access to data sources in all regions. Therefore, the actual number of prehospital deaths is unclear. Additionally, although such data could provide valuable insight for injury prevention, forensic institutes are currently not permitted to share this information due to privacy protection regulations. Fourth, the registry continuously works to improve data quality and regularly updates the database content as needed, but any change to the database must be made through the developer. Such changes incur considerable costs to the NTR and often take time to implement. Finally, the registry only collects data on patients who are severely or potentially severely injured. Therefore, it has been suggested that, to obtain a complete overview of the burden of accidents and injuries, a national injury registry should be established [74, 75].

The registry aims to establish a more robust system for collecting rehabilitation data. We also recommend implementing a regular international revision process to update the Utstein Trauma Template data variables. Additionally, a European Trauma Core Dataset should be developed, refining objectives, data variables, and clinical indicators essential for international comparisons of trauma care and systems.

## Conclusions

The NTR has been successfully implemented across all trauma-receiving hospitals in Norway, with all hospitals submitting data. Certified data registrars at each hospital ensure systematic data collection, and most hospitals monitor undertriage, although some currently lack the resources to identify all undertriaged patients. The registry demonstrates excellent individual-level coverage, with high completeness and accuracy. The registry will continue to enhance data completeness and coverage, and it is expected to play an increasingly significant role in monitoring patient safety, evaluating the quality of the trauma system, and facilitating research initiatives.

## Electronic supplementary material

Below is the link to the electronic supplementary material.

Supplementary Material 1

## Data Availability

No datasets were generated or analysed during the current study.

## Abbreviations

EMCC: Emergency Medical Communication Centre
EQ-5D-5L: European Quality of Life 5-Dimension 5-Level
HEMS: Helicopter Emergency Medical Services
IQR: Interquartile range
ISS: Injury Severity Score
NISS: New Injury Severity Score
NTR: Norwegian Trauma Registry
PROMs: Patient Reported Outcome Measures
