# Supplementary material for: The Norwegian national trauma registry: development process and essential data insights
Source: Scand J Trauma Resusc Emerg Med. 2025 May 1;33:78. doi: 10.1186/s13049-025-01390-7 (PMC12044736; doi:10.1186/s13049-025-01390-7)
Supplement: Supplementary file 1 — Supplementary Material 1 [file 13049_2025_1390_MOESM1_ESM.docx]

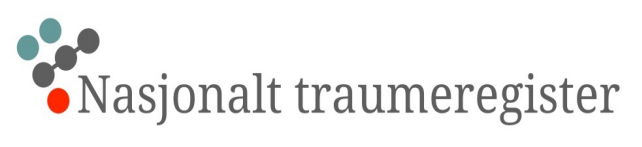


**13 PROM**

**13.2 Additional Questions:**

**1) Did you have any long-term (at least one year) illness, injury, or disorder of a physical or psychological nature that resulted in reduced functionality in your daily life prior to the accident?**

**Data type:**

Nominal

**Data variable categories:**

1 = Yes

2 = No

**Abbreviated field name:**

LangvarigSykdom

**If YES:** To what extent would you say your functions were impaired?

| **Mobility impairment**  1 = Not impaired  2 = Slightly impaired  3 = Moderately impaired  4 = Severely impaired | **Vision impairment**  1 = Not impaired 2 = Slightly impaired 3 = Moderately impaired 4 = Severely impaired | **Hearing impairment**  1 = Not impaired  2 = Slightly impaired  3 = Moderately impaired  4 = Severely impaired | **Impairment due to physical illness**  1 = Not impaired  2 = Slightly impaired  3 = Moderately impaired  4 = Severely impaired | **Impairment due to mental illness**  1 = Not impaired  2 = Slightly impaired  3 = Moderately impaired  4 = Severely impaired |
| --- | --- | --- | --- | --- |
| **Data type**  Ordinal | **Data type**  Ordinal | **Data type**  Ordinal | **Data type**  Ordinal | **Data type**  Ordinal |
| **Data variable categories**  Numeric | **Data variable categories**  Numeric | **Data variable categories**  Numeric | **Data variable categories**  Numeric | **Data variable categories**  Numeric |
| **Abbreviated field name**  Bevegelseshemmet | **Abbreviated field name**  NedsattSyn | **Abbreviated field name**  NedsattHorsel | **Abbreviated field name**  KroppsligSykdom | **Abbreviated field name**  PsykiskSykdom |

**2) Were you employed or in education at the time of the accident?**

**Data type:**

Nominal

**Data variable categories:**

1 = No

2 = Yes

**Abbreviated field name:**

JobbUtdanning

**3. Have you returned to work or education since the injury?**

**Data type:**

Nominal

**Data variable categories:**

1 = No

2 = Yes

**Abbreviated field name:**

TilbakeJobbUtdanning

**4. What is your current employment status?**

**Data type:**

Nominal

**Data variable categories:**

1 = Paid employment

2 = Self-employed

3 = Education/military service

4 = Unemployed/on leave

5 = Retired/on disability benefits

**Abbreviated field name:**

1. **As a result of the injury, have you received healthcare treatment within the past six months?**

**Data type:**

Nominal

**Data variable categories:**

1 = No

2 = Yes

**Abbreviated field name:**

MottattBehandling

1. **NRS/VAS scale:** **The patient is asked to rate their current level of pain.**


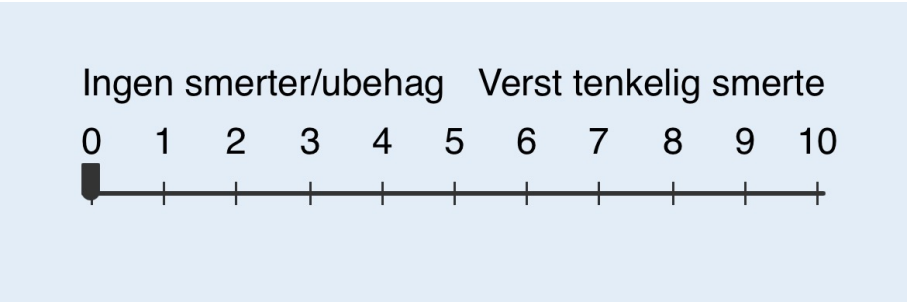


**Data variable definition:**

Visual Analogue Scale (VAS)

**Data type:**

Ordinal

**Data variable values:**

Format: 0–10

**Abbreviated field name:**

PasientsvarVasSmerte
